# Street Medicine: Barrier Considerations for Healthcare Providers in the U.S.

**DOI:** 10.7759/cureus.38761

**Published:** 2023-05-09

**Authors:** Emmanuel Tito

**Affiliations:** 1 Internal Medicine, Western Michigan University Homer Stryker M.D. School of Medicine, Kalamazoo, USA

**Keywords:** medical liability, preventive medication, medicine in resource limited areas, medical care, safety and health, medication dispensing, underserved care, electronic medical records (emr), homelessness, street medicine

## Abstract

In the last decade, a movement known as “street medicine” has emerged. It is a relatively new medical field in which healthcare providers deliver medical care to homeless populations outside of traditional healthcare facilities, on the streets, and in various settings where unsheltered people live. Physicians essentially visit people living in camps, along riverbanks, in alleys, and abandoned buildings to provide medical care. During the pandemic, street medicine in the U.S. was often the first line of defense for people living on the streets. As the practice of street medicine continues to grow and expand across the country, there is an increasing demand to standardize patient care delivered outside traditional healthcare facilities.

## Editorial

Street medicine is the practice of medicine in areas outside of the traditional hospital. Typically, it involves medical care for individuals that are experiencing homelessness or living in poverty. These individuals may not have access to traditional healthcare services and thus healthcare services are brought to them. This practice can occur in shelters, directly on the street, in designated encampments, and places outside the walls of a traditional hospital or clinic. Street medicine is assembled by a variety of healthcare providers that include, but are not limited to, physicians, nurse practitioners, and physician assistants [1]. The origins of street medicine in the U.S. date back to 1992, when Jim Withers, a faculty attending physician at the University of Pittsburgh Medical Center Mercy Hospital, provided free healthcare to those in need in his community. Since then, street medicine has evolved nationally and has become an institution that provides communities and clinicians with expert training, guidance, and support to develop and grow their street medicine programs [1].

One of the challenges for people experiencing homelessness is accessing health care as a result of several barriers, both structural and economic [2]. Homeless men and women have a life span nearly 30 years shorter than their housed counterparts, and less than 10% have a primary care physician [3]. These individuals often seek medical care when their illness is already advanced. Unfortunately, this results in undue morbidity and mortality in these underserved populations. There is growing evidence that the chronic street homeless population experiences a disproportionate amount of cost due to prolonged hospitalizations and recurrent emergency visits [4]. It also puts undue strain on healthcare resources and raises healthcare costs.

Advocates of street medicine argue that it reduces health inequities and stigma among excluded populations. They also highlight that it allows early interventions and easier access to healthcare services. Street medicine programs have been shown to produce significant cost savings and represent a cost-effective delivery model that improves health outcomes in underserved populations [4,5]. The increased demand for street medicine has been in part due to its preventive nature, reducing more serious and costly complications thus decreasing healthcare-associated costs.

Despite its numerous benefits, opponents are concerned about the lack of oversight and regulation to control this practice. The challenges of street medicine are lack of clear organizational structures and chain of command; lack of standard referral procedures and protocols to ensure timely access to continuity primary care; unavailability of electronic medical records (EMR) and data tracking system on the streets; liability coverage issues for street medicine providers; medication dispensing and safety guidelines.

Street medicine services are considered a link between clinical and community settings to serve medically underserved individuals who are disproportionately affected by many illnesses due to disparity in social determinants of health. In an effort to have a clear organizational structure and chain of command, several authors promoted the use of a system such as the Incident Command Structure developed by state and local governments for disaster readiness and response [6]. A street medicine team can consist of an incident commander who is the attending physician and four team leaders: finance chair; logistics chair (supply chain, volunteer recruitment, and transportation); operation manager; and planning manager. This change allows adequate coordination of efforts with local forces and critical community partners. According to Los Angeles Christian Health Centers, street medical teams typically consist of a primary care provider, a medical assistant, and a registered nurse [7]. The medical team will outreach to local partners to provide care for the unsheltered. Concerns about street medicine’s organizational structure continue to rise. Several individuals are often involved in the operations on the street without a distinct role. Medical students, residents, clinicians, nurses, and social workers are often seen on the streets. Not all parties involved in the preparedness and response team need to be on the street. In fact, the majority of the work is done behind the scenes.

Another issue that arises with promoting street medicine is the lack of standard referral procedures and protocols to ensure that unsheltered patients are followed up in continuity clinics. Street medicine programs tend to be quick at addressing the immediate medical needs of the patients encountered on the streets without creating policies and procedures first. Basic protocols should be established and reviewed by legal counsels and risk management offices of the institutions involved. Current protocols suggested by the Agency for Healthcare Research and Quality include homeless outreach initiatives, interactions with the media, and patient follow-ups [4]. Academic institutions or teaching hospitals must develop alliances with local institutions that serve unsheltered populations. These local agencies tend to know their needs and behaviors and could assist with scheduling follow-up appointments. Allies can include the local police, the Department of Health and Human Services, homeless shelters, housing authorities, and substance use counseling services. These partnerships enhance patient care and strengthen the physician-patient relationship. From a legal perspective, it is necessary to have written regulations and guidelines about the responsibilities of each medical team member, especially volunteers, students, healthcare workers, and resident doctors, to avoid anyone working above their level of education or expertise. This also ensures providers are aware of their scope of practice.

Many see street medicine as a solution to the provision of health care to the unsheltered homeless to ease the burden of hospitals. The current practice of street medicine programs consists of documenting doctor-patient encounters and following up with patients to support compliance with any aftercare [2-4]. Street medicine must be held to the same standard as any healthcare facility delivering medical care, and that includes clinical record keeping. State and federal statutes have laws in place to maintain Health Insurance Portability and Accountability Act (HIPPA) compliance and to ensure that patients’ privacies are protected. Indeed, street medicine is practiced in a public environment, but the medical team must file documentation properly, either with patient records in the file box or saved electronically in the EMR system. Street encounters can be documented in the home institution’s EMR. There are also reasonably priced, cloud-based mobile electronic medical records that can be individualized [2,4]. Integrating medical records in health electronic records allows for improvement in care coordination through quality improvement projects [7,8].

One concern that arises in the conversation about expanding street medicine is the question of liability coverage for healthcare providers. When delivering care on the streets, or treating patients in homeless shelters, healthcare providers are often unfamiliar with patient histories and may be treating them for conditions that are not within the provider’s regular scope of practice. Diagnostic capabilities and equipment may be limited. When treating patients with mental disorders or substance use disorders, the healthcare provider may not have direct access to behavioral health specialists to assist in decision-making [8].

Since physicians often provide free or reduced-cost care, how does it affect malpractice coverage? Do Good Samaritan laws provide protection from legal action on the streets [8]? These are just some of the questions that need to be addressed prior to advancing the practice of street medicine. Street medicine teams typically rely on point-of-care testing, ultrasound, and diagnostic algorithms given that sophisticated laboratory and advanced diagnostic tests may not be available for financial or practical reasons. Healthcare providers should also be concerned about ramifications related to incomplete treatment when they provide care on the streets [8]. What kind of liability (if any) do healthcare providers incur by diagnosing a patient and recommending treatment plans in the absence of the standard of care diagnostic tests? The Good Samaritan law is a legal protection designed to encourage individuals to aid in emergency situations without fear of being sued for their actions or inactions. This law empowers physicians to act in emergencies without fear of legal repercussions. However, the law also only protects providers from claims of negligence. Therefore, if the provider is accused of purposeful harm or acting outside the scope of their training, they could still be subject to legal action. Furthermore, the law varies from state to state. For example, Michigan determined that surgeons who were not on call but contacted by the emergency department to help a patient were not held liable for poor outcomes due to good Samaritan protection. New Jersey on the other hand found that “the protection of the Good Samaritan Act stops at the door of the hospital.” Meaning it does not expand or cover the practices of street medicine. This law serves as a blanket to cover those who want to participate in street medicine, however, it is on the provider to understand their limitations and their scope of practice for their respective state [9].

Most states provide limited liability or immunity for physicians who volunteer their professional services, and others subsidize the purchase of malpractice insurance [8]. At the federal level, The Volunteer Protection Act of 1997 (VPA) was enacted to provide immunity to individuals who perform volunteer work for nonprofit organizations or governmental entities from civil liability injuries they cause by acts of negligence while volunteering. The VPA establishes a minimum level of protection for volunteers and preempts conflicting state law unless the state law provides greater protection [4]. Current federal law protections are designed for healthcare professionals volunteering for qualifying free clinics. These protections under which they apply, vary widely by state. They may not be applicable to the practice of street medicine. State and federal laws for street medicine need to be clearly defined if an adverse event occurs.

Storing and dispensing medication are major challenges in street medicine. Standard policies and procedures regarding medication storage and dispensing are non-existent. Medications in backpacks are at risk of being stored outside the recommended temperature. The expiration date may not be adequately monitored. They are also at risk of being accessed by non-qualified prescribers. Furthermore, there has been no consensus on dispensing controlled substances (opiates, benzodiazepines) and psychotropic medications (mood stabilizers, antidepressants, antipsychotics, stimulants for attention-deficit/hyperactivity disorder). It is imperative to have a formulary medication list, tamper-resistant packaging, and dispensing logs. Prescribers must also practice within the scope of their license and compliance with their institution credentials. Medications can be dispensed in tamper-evident packaging or pill bottles unless a waiver is signed by the patient and documented in the clinical record chart. If medications are unit dosed, they may be dispensed in a plastic zip lock bag with a label fixed onto the bag [7-9]. It is important that medications be dispensed with the local street medicine label with all the proper information filled on the label sticker. Dispensed medications can be documented on a medication dispense log and then later reported on the EMR if not available on the site. Pharmacy can assist with establishing safe medication storage and handling processes. An established protocol can facilitate continuity of care, especially for subsequent visits.

The benefits of street medicine are recognized in the medical community, and its capability to deliver medical care to several patients who may not have access to healthcare. There is a need to provide a comprehensive framework that establishes guidelines to address street medicine at the national and international levels while allowing institutions to incorporate policies that meet the healthcare needs of their communities. Street medicine must not be practiced in a way that would diminish patient-centered care or the quality of care being provided to patients. The standard of care provided through street medicine should be comparable to that of care provided when the healthcare provider and patient are face-to-face. The practice of street medicine must have policies and regulations in place to ensure patient privacy and to ensure the safety of healthcare professionals. Fears of liability should not discourage providers from practicing street medicine. Rather, they should advocate for the existence of state immunity statuses and policies on liability reforms. 
